# The unique association between the level of peripheral blood monocytes and the prevalence of diabetic retinopathy: a cross-sectional study

**DOI:** 10.1186/s12967-020-02422-9

**Published:** 2020-06-22

**Authors:** Heng Wan, Yan Cai, Yuying Wang, Sijie Fang, Chi Chen, Yi Chen, Fangzhen Xia, Ningjian Wang, Minghao Guo, Yingli Lu

**Affiliations:** 1 Institute and Department of Endocrinology and Metabolism, Shanghai Ninth People’s Hospital, Shanghai Jiao Tong University School of Medicine, Shanghai, 200011 China; 2 Department of Endocrinology, The Fifth Affiliated Hospital of Kunming Medical University, Yunnan Honghe Prefecture Central Hospital (Ge Jiu People’s Hospital), Yunnan, China; 3 Department of Ophthalmology, Shanghai Ninth People’s Hospital, Shanghai Jiao Tong University School of Medicine, Shanghai, China

**Keywords:** Monocytes, Diabetic retinopathy, Leukocyte characteristics, Cardiovascular and cerebrovascular diseases, Diabetic kidney disease

## Abstract

**Objective:**

The attraction and influx of monocytes into the retina has been considered a critical step in the development of diabetic retinopathy (DR). However, large population studies about the association between peripheral blood monocyte levels, an inexpensive and easily measurable laboratory index, and DR are limited. Thus, we aimed to investigate the association between peripheral blood monocyte levels and DR.

**Methods:**

A total of 3223 participants out of 3277 adults with diabetes were enrolled from seven communities in China in this cross-sectional survey. Participants underwent several medical examinations, including the measurement of anthropometric factors, blood pressure, routinely analyzed leukocyte characteristics, glucose, lipid profiles, urine albumin/creatinine ratio and fundus photographs.

**Results:**

The prevalence of DR among the participants in the highest quartile of peripheral blood monocyte levels significantly decreased by 41% (OR 0.59; 95% CI 0.43, 0.81) compared with the participants in the first quartile (*P* for trend < 0.05). However, there were no associations between the monocyte level and the prevalence of cardiovascular and cerebrovascular diseases (CVD) and diabetic kidney disease (DKD) (both *P* for trend > 0.05). Associations between leukocyte, neutrophil and lymphocyte levels and DR were also not found (all *P* for trend > 0.05). These associations were all fully adjusted for age, sex, education status, duration of diabetes history, current smoking, BMI, HbA1c, dyslipidemia, systolic blood pressure and insulin therapy.

**Conclusion:**

Decreased peripheral blood monocyte levels were associated with increased odds of DR after adjusting for potential confounders in diabetic adults. However, causation remains to be demonstrated.

## Introduction

Diabetes mellitus (DM) has become a global epidemic with serious morbidity and mortality. Recent studies suggested that of the 600 million people with DM worldwide by 2040, 400 to 500 million will live in low- and middle-income countries, and half will present with diabetic microvascular complications [1, 2]. Diabetic retinopathy (DR), one of the diabetic microvascular complications, remains one of the leading causes of blindness, and much of the blindness from DR is preventable with early detection and treatment [3]. Epidemiological investigations have reported that approximately 1 in 3 persons with DM has DR, and 1 in 10 has proliferative DR or diabetic macular edema [4, 5]. However, using ophthalmologists to screen every diabetic patient for DR is not feasible because most persons diagnosed with DM are receiving treatment from endocrinologists, and in developing countries such as China, most endocrinologists do not have ophthalmic examination equipment such as wide-field retinal photography and optical coherence tomography [1, 6]. Thus, considerable attention should be paid to the development of feasible screening for DR, especially for endocrinologists in developing countries.

Plenty of evidence has suggested that retinal inflammation plays a pivotal role in the pathogenesis of DR [7–9]. Elevated inflammatory mediator levels resulting from the accumulation of advanced glycation end products may cause persistent chronic inflammation of the diabetic retina, which would bring about leukocyte activation, adhesion to the vascular endothelium and extravasation into retinal tissues [10, 11]. Monocytes, one of the major leukocyte subtypes, have been considered as an inflammatory biomarker [12]. Animal studies have revealed the influx of considerable perivascular monocytes into retinal tissues, and the retinal pigment epithelium serves a parallel role as a gateway for monocyte trafficking to the retina [13, 14]. One recent human study reported that the level of monocyte chemoattractant protein-1 (MCP-1), which regulates monocyte chemotaxis and modulates inflammatory processes, was significantly increased in the plasma of patients with DR [15]. However, to the best of our knowledge, no studies have evaluated the associations of monocyte levels with DR in a large number of people.

In addition, in daily clinical practice, the measurement of routinely analyzed leukocyte characteristics, which includes monocyte level, is more common than many other inflammatory markers, such as interleukin and MCP-1, because of the costs and technical difficulties in measuring the inflammatory markers. If the hypothesis that monocyte levels are associated with the prevalence of DR is demonstrated, clinicians could conduct DR screening for diabetic patients more feasibly, and this may provide evidence to support the prevention and treatment of DR by targeting monocyte trafficking. Thus, in the present study, we aimed to investigate the associations between the level of monocytes and the prevalence of DR in adults with DM.

## Materials and methods

### Study design and participants

The present cross-sectional study was designed in 2018. We enrolled study participants from the registration platform of the healthcare center in seven communities in Huangpu and Pudong District, Shanghai, China, from May 2018 to August 2018. The enrolled citizens were ≥ 18 years old and had lived in the current area for ≥ 6 months. A total of 3277 subjects with diabetes received an examination. Participants who were missing routinely analyzed leukocyte characteristics results (n = 11) were excluded. We then excluded participants who had received treatment, including laser photocoagulation or the use of intravitreal anti-vascular endothelial growth factor inhibitors (n = 43), in the past week. Finally, the number of participants who were involved in the analyses for the association between monocyte levels and the prevalence of diabetic retinopathy was 3223.

The study protocol conformed to the ethical guidelines of the 1975 Declaration of Helsinki as reflected in the priori approval by the Ethics Committee of Shanghai Ninth People’s Hospital, Shanghai Jiao Tong University School of Medicine. Written consent was obtained from all the participants in our study.

### Measurements

A questionnaire about demographics, medical history, family history and lifestyle factors was completed by the same trained personnel as in our previous studies [16, 17] during the interview. Clinical examination measurements, including height, weight and blood pressure, were performed according to the same standard protocol as before [18, 19]. Body mass index (BMI) was calculated as the weight in kilograms divided by the height in meters squared. We defined current smoking as having smoked at least 100 cigarettes in the lifetime and smoking cigarettes currently [20].

Overnight fasting blood (at least 8 h of fasting) was obtained between 6:00 and 9:00 am, refrigerated immediately and sent to a central laboratory for measurement in 2 h. Routinely analyzed leukocyte characteristics, including leukocyte, neutrophil, lymphocyte, and monocyte levels, were measured with the XS-800i (Sysmex, Japan). Fasting plasma glucose (FPG), serum creatinine, total cholesterol, triglycerides, high (HDL) and low-density lipoprotein (LDL) were detected with the Beckman Coulter AU 680 (Brea, USA). Glycated hemoglobin (HbA1c) was tested using high-performance liquid chromatography with the MQ-2000PT (Shanghai, China). Morning urine samples were collected and immediately placed in the refrigerator; the levels of urine albumin and creatinine were measured with the Beckman Coulter AU 680 (Brea, USA), and then the urine albumin/creatinine ratio (ACR) was calculated. Participants were diagnosed with DR by a remote reading conducted by ophthalmologists using retinal fundus photography with a Topcon TRC-NW400 Non-Mydriatic Retinal Camera (Oakland, USA), as before [19, 21].

### Outcome definition

The definition of dyslipidemia was total cholesterol ≥ 6.22 mmol/L (240 mg/dL), triglycerides ≥ 2.26 mmol/L (200 mg/dL), LDL ≥ 4.14 mmol/L (160 mg/dL), HDL < 1.04 mmol/L (40 mg/dL), or a self-reported previous diagnosis of hyperlipidemia, according to the modified National Cholesterol Education Program-Adult Treatment Panel III. The outcome CVD was defined as a previous diagnosis with coronary heart disease, stroke, or peripheral arterial disease and was recorded in the registration platform as before [22, 23]. The estimated glomerular filtration rate (eGFR) was calculated by the Chronic Kidney Disease Epidemiology Collaboration equation for “Asian origin”. The definition of DKD was ACR ≥ 30 mg/g and/or eGFR < 60 mL/min per 1.73 m^2^, as suggested by the American Diabetes Association statement [24].

The DR classification was as follows: DR stage 0 was defined as no abnormalities; DR stage 1 to 3 nonproliferative DR (NPDR) was defined as intraretinal microaneurysms, hemorrhages, venous beading, prominent microvascular abnormalities; and DR stage 4 proliferative DR (PDR) was defined as neovascularization or vitreous/preretinal hemorrhages in accordance with the “Global Diabetic Retinopathy Project Group” [25].

### Statistical analysis

IBM SPSS Statistics, Version 22 (IBM Corporation, Armonk, NY, USA) was used in the current analysis. A *P* value (two sided) < 0.05 indicated significance. Continuous variables were expressed as the mean ± SD, and categorical variables were expressed as percentages (%) or medians (interquartile range). One-way ANOVA or Student t-test and the Chi square test were used for the comparison of continuous and categorical variables, respectively. Monocyte levels were divided into quartiles.

A regression analysis was used to detect the associations between routinely analyzed leukocyte characteristic levels and diabetic complications. Data were summarized as odds ratios or regression coefficients (95% CIs). The associations of monocyte, leukocyte, neutrophil and lymphocyte levels with the prevalence of DR, CVD and DKD were tested by binary logistic regression analyses. The cutoff value of the monocyte level with the largest Youden index for predicting DR was tested by receiver operating characteristic (ROC) curve analysis.

Sensitivity analyses were performed. To minimize the impact of sample selection, the associations between the monocyte level quartiles and the prevalence of DR among the participants without PDR were evaluated in Additional file 1: Table S1. In addition, to reduce the impact of the exclusion criteria on the associations, we also investigated the associations between the monocyte level quartiles and the prevalence of DR among all 3266 participants who had reliable and complete medical records in Additional file 1: Table S2. The associations between the C-reactive protein (CRP) level and diabetic complications are shown in Additional file 1: Table S3.

## Results

The general and sociodemographic characteristics of the participants in the study are shown in Table 1. A total of 3223 diabetic participants with a mean age of 67 years old (SD 8, min 23, max 99) were involved in the final analyses. A total of 2709 (84.1%) participants were diagnosed without DR; 334 (10.4%) participants were diagnosed with DR stage 1; 166 (5.2%) participants were diagnosed with DR stage 2; 12 (0.4%) participants were diagnosed with DR stage 3; and 2 (0.1%) participants were diagnosed with DR stage 4. The participants were divided into two groups based on whether they had DR. Compared with the participants without DR, the duration of diabetes, FPG, HbA1c, ACR, systolic blood pressure level, and the prevalence of DKD and CKD were significantly higher in patients with DR (all *P* < 0.05). Although no differences in leukocyte, lymphocyte and neutrophil levels were found between the two groups, the monocyte level was significantly lower in patients with DR than in participants without DR (*P* < 0.05).

**Table 1:** General characteristics of the participants by diabetic retinopathy

Characteristic	DR−	DR+	*P*
*N*	2709	514	–
Age, years	67.11 ± 8.48	66.96 ± 7.92	0.710
Men, %	46.1	47.5	0.559
Duration of diabetes, years	8 (3,15)	10 (5,18)	< 0.001
Current smoking, %	17.6	16.4	0.518
Beyond high school education, %	52.1	53.4	0.577
BMI, kg/m^2^	24.89 ± 3.59	25.29 ± 3.63	0.019
FPG, mmol/L	7.73 ± 2.34	8.25 ± 2.84	< 0.001
HbA1c, %	7.42 ± 1.33	7.78 ± 1.53	< 0.001
Total cholesterol, mmol/L	5.12 ± 1.19	5.17 ± 1.21	0.343
Triglycerides, mmol/L	1.56 (1.11, 2.24)	1.43 (1.08, 2.09)	0.498
HDL, mmol/L	1.21 ± 0.3	1.20 ± 0.29	0.766
LDL, mmol/L	3.16 ± 0.84	3.22 ± 0.86	0.176
ACR, mg/g	12.0 (7.0, 26.0)	16.0 (9.0, 42.5)	< 0.001
eGFR, mL/min per 1.73 m^2^	91.56 ± 16.90	92.69 ± 16.76	0.161
Systolic blood pressure, mmHg	144.11 ± 19.51	148.40 ± 20.30	< 0.001
Diastolic blood pressure, mmHg	78.63 ± 10.88	78.63 ± 10.80	0.999
DKD, %	24.7	34.9	< 0.001
CVD, %	36.1	41.0	0.035
Dyslipidemia, %	62.9	62.8	0.866
Leukocyte (× 10^9^/L)	6.44 ± 1.68	6.38 ± 1.62	0.480
Lymphocytes (× 10^9^/L)	2.09 ± 0.65	2.09 ± 0.80	0.923
Neutrophils (× 10^9^/L)	3.77 ± 1.28	3.74 ± 1.17	0.608
Monocytes (× 10^9^/L)	0.38 ± 0.12	0.36 ± 0.11	0.013
insulin therapy, %	15.8	26.6	< 0.001

The data are summarized as the mean ± SD for continuous variables with a distribution, the median (interquartile ranges) for continuous variables with a skewed distribution or a numerical proportion for categorical variables

*CVD* cardiovascular and cerebrovascular disease, *DKD* diabetic kidney disease, *DR* diabetic retinopathy, *BMI* body mass index, *FPG* fasting plasma glucose, *HbA1c* glycated hemoglobin, *HDL* high-density lipoprotein, *LDL* low-density lipoprotein, *ACR* albumin to creatinine ratio, *eGFR* estimated glomerular infiltration rate

The characteristics of the participants by the monocyte level quartiles are shown in Table 2. Compared with the participants in the lowest monocyte level quartile, those in the highest quartile were more likely to have an older age; be male and a current smoker; have higher BMI, FPG, HbA1c, triglycerides, urine ACR, diastolic blood pressure and prevalence of dyslipidemia; and have lower total cholesterol, LDL, HDL and eGFR (all *P* for trend < 0.05). Although the prevalence of CVD and DKD among the participants in the highest monocyte level quartile were marginally and significantly higher than those in the lowest quartile, respectively, the prevalence of DR among the participants in the highest monocyte level quartile was marginally lower without adjusting for any potential confounders.

**Table 2:** Characteristics of the participants by monocyte level quartiles

Characteristic	Monocytes (× 10^9^/L)				*P* for trend
	Quartile 1	Quartile 2	Quartile 3	Quartile 4	
	(≤ 0.29)	(> 0.29, ≤ 0.36)	(> 0.36, ≤ 0.44)	(> 0.44)	
*N*	818	833	772	800	–
Age, years	66.36 ± 8.08	67.14 ± 8.02	67.60 ± 8.59	67.29 ± 8.85	0.014
Men, %	28.1	38.5	54.0	65.5	< 0.001
Duration of diabetes, years	8 (3, 15)	9 (3, 15)	10 (4, 16)	10 (4, 15)	0.105
Current smoking, %	8.6	11.7	18.6	31.0	< 0.001
Beyond high school education, %	49.3	53.2	54.4	52.5	0.172
BMI, kg/m^2^	24.43 ± 3.78	24.75 ± 3.47	25.24 ± 3.47	25.44 ± 3.59	< 0.001
FPG, mmol/L	7.46 ± 2.33	7.76 ± 2.39	8.02 ± 2.56	8.02 ± 2.44	< 0.001
HbA1c, %	7.16 ± 1.25	7.41 ± 1.33	7.65 ± 1.44	7.70 ± 1.39	< 0.001
Total cholesterol, mmol/L	5.27 ± 1.26	5.11 ± 1.16	5.13 ± 1.15	4.99 ± 1.18	< 0.001
Triglycerides, mmol/L	1.39 (1.00, 1.94)	1.46 (1.06, 2.18)	1.66 (1.17, 2.40)	1.65 (1.21, 2.32)	< 0.001
HDL, mmol/L	1.30 ± 0.32	1.23 ± 0.29	1.18 ± 0.28	1.12 ± 0.27	< 0.001
LDL, mmol/L	3.24 ± 0.88	3.16 ± 0.85	3.19 ± 0.81	3.10 ± 0.82	0.002
ACR, mg/g	11 (7, 22)	12 (7, 28)	13 (7, 32)	15 (8, 32)	0.026
eGFR, mL/min per 1.73 m^2^	93.20 ± 15.96	93.25 ± 15.10	90.73 ± 16.96	89.64 ± 19.08	< 0.001
Systolic blood pressure, mmHg	144.01 ± 20.60	145.00 ± 19.38	145.39 ± 19.64	144.86 ± 19.14	0.352
Diastolic blood pressure, mmHg	77.24 ± 10.05	78.01 ± 10.79	79.55 ± 11.16	79.81 ± 11.28	< 0.001
Dyslipidemia, %	54.6	63.3	65.9	67.9	< 0.001
CVD, %	34.6	35.9	38.5	38.6	0.056
DKD, %	20.4	25.9	28.7	30.6	< 0.001
DR, %	16.9	16.7	16.8	13.4	0.072
Insulin therapy, %	13.2	15.8	20.3	21.5	< 0.001

The data are summarized as the mean ± SD for continuous variables with a normal distribution, the median (interquartile ranges) for continuous variables with a skewed distribution, or a numerical proportion for categorical variables

*P* for trend was calculated by regression tests

*BMI* body mass index, *FPG* fasting plasma glucose, *HbA1c* glycated hemoglobin, *HDL* high-density lipoprotein, *LDL* low-density lipoprotein, *CVD* cardiovascular and cerebrovascular disease, *DKD* diabetic kidney disease, *DR* diabetic retinopathy, *ACR* albumin to creatinine ratio, *eGFR* estimated glomerular infiltration rate

**Fig. 1:**
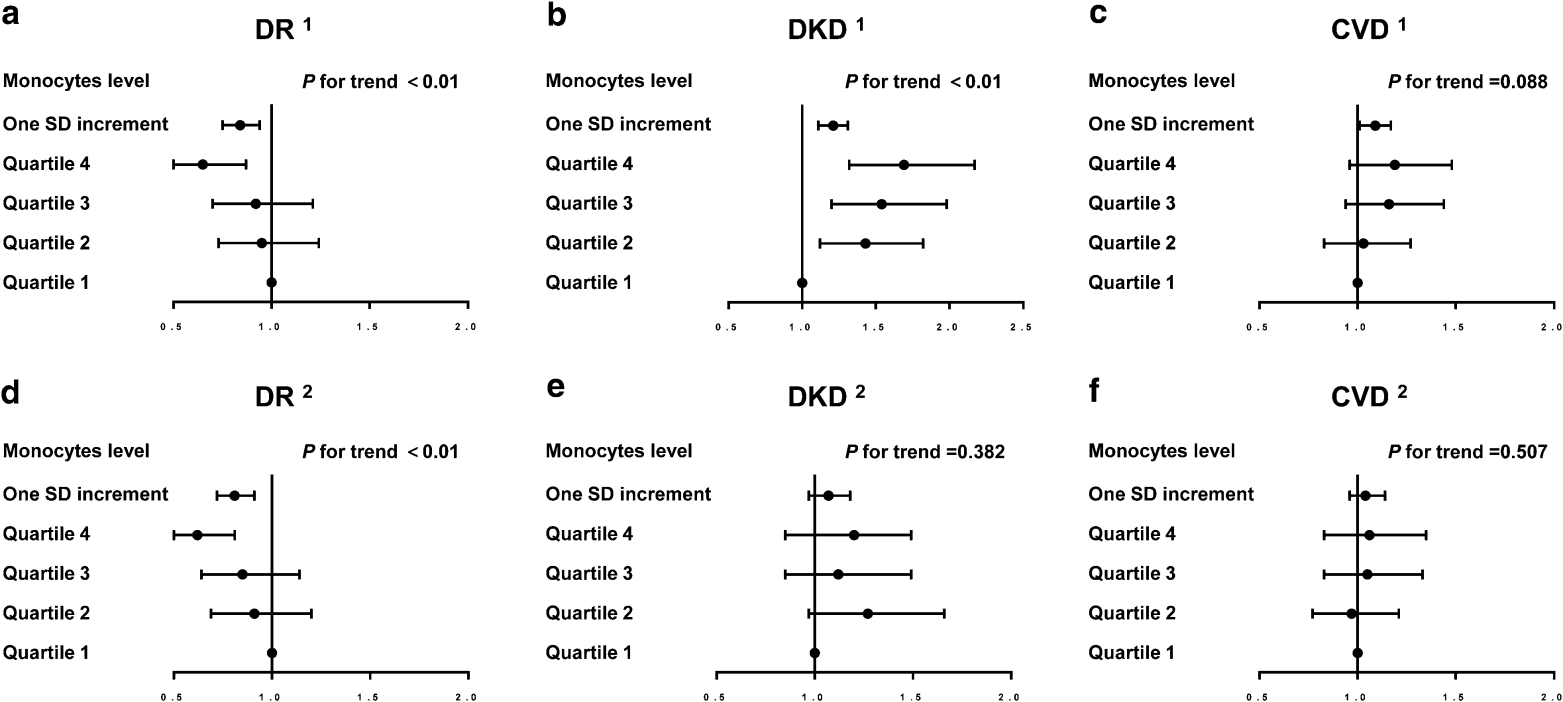
Associations between monocyte levels and diabetic complications. **a** Monocytes and DR; **b** monocytes and DKD; **c** monocytes and CVD; **d** monocytes and DR, adjusting for the full model; **e** monocytes and DKD, adjusting for the full model; **f** monocytes and CVD, adjusting for the full model. ^1^The model was adjusted for age, sex, and duration of diabetes. ^2^The model was adjusted for age, sex, duration of diabetes, education status, current smoking, BMI, HbA1c, dyslipidemia, systolic blood pressure and insulin therapy. *CVD* cardiovascular and cerebrovascular disease, *DKD* diabetic kidney disease, *DR* diabetic retinopathy, *BMI* body mass index, *HbA1c* glycated hemoglobin

We evaluated the associations between monocyte levels and diabetic complications in Fig. 1 and found that elevated monocyte levels were significantly associated with a decreased prevalence of DR after adjusting for potential confounders; however, no significant associations were found between monocyte levels and the prevalence of CVD and DKD after full adjustment for potential confounders. Compared with the first quartile of the monocyte level, the odds of having DR was significantly decreased by 27% for participants in the highest quartile after adjusting for age, sex and duration of diabetes (*P* for trend < 0.01). One SD increase in monocyte levels was also significantly related to the prevalence of DR (OR 0.84; 95% CI 0.75, 0.94) (*P* < 0.05). After adjusting for age, sex, education status, duration of diabetes, current smoking, BMI, HbA1c, dyslipidemia, systolic blood pressure and insulin therapy, the associations remained. The prevalence of DR among the participants in the highest quartile of peripheral blood monocyte levels significantly decreased by 41% (OR 0.59; 95% CI 0.43, 0.81) compared with the participants in the first quartile in the fully-adjusted model. Although the elevated monocyte level was significantly associated with an increased prevalence of DKD after adjusting for age, sex and duration of diabetes (*P* for tend < 0.001), no association was found between the monocyte level and DKD after adjusting for age, sex, education status, duration of diabetes, current smoking, BMI, HbA1c, dyslipidemia, systolic blood pressure and insulin therapy. Furthermore, we also found no associations between the monocyte level and the prevalence of CVD in either the initial model adjusting for age, sex and duration of diabetes or in the full model adjusting for age, sex, education status, duration of diabetes, current smoking, BMI, HbA1c, dyslipidemia, systolic blood pressure and insulin therapy.

**Fig. 2:**
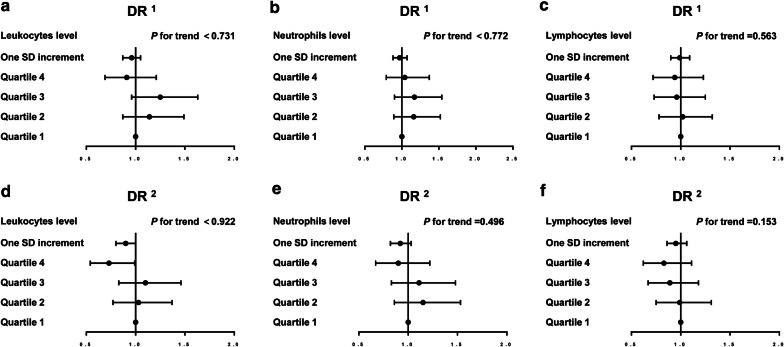
Associations between leukocyte, neutrophil and lymphocyte levels and DR. **a** Leukocytes and DR; **b** neutrophils and DR; **c** lymphocytes and DR; **d** leukocytes and DR, adjusting for the full model; **e** neutrophils and DR, adjusting for the full model; **f** lymphocytes and DR, adjusting for the full model. ^1^The model was adjusted for age, sex, and duration of diabetes. ^2^The model was adjusted for age, sex, duration of diabetes, education status, current smoking, BMI, HbA1c, dyslipidemia, systolic blood pressure and insulin therapy. *DR* diabetic retinopathy, *BMI* body mass index, *HbA1c* glycated hemoglobin

In addition, associations between leukocyte, neutrophil and lymphocyte levels and DR were detected. Figure 2 shows that the prevalence of DR was not associated with the leukocyte, neutrophil and lymphocyte levels. In the initial model adjusting for age, sex and duration of diabetes, associations of the prevalence of DR with the leukocyte, neutrophil and lymphocyte level quartiles were not found (all *P* for trend > 0.05). After adjusting for age, sex, education status, duration of diabetes history, current smoking, BMI, HbA1c, dyslipidemia, systolic blood pressure and insulin therapy, there were also no associations between the leukocyte, neutrophil and lymphocyte levels and the prevalence of DR (all *P* for trend > 0.05). The monocyte level cutoff value with the largest Youden index of was 0.405 (× 10 9/L), with a sensitivity of 36.4% and specificity of 70.5% (*P* < 0.05).

Sensitivity analyses were performed. Among the participants without proliferation, an elevated monocyte level was significantly associated with a decreased prevalence of DR in both the initial model adjusting for age, sex and duration of diabetes and in the full model adjusting for age, sex, education status, duration of diabetes, current smoking, BMI, HbA1c, dyslipidemia, systolic blood pressure and insulin therapy (Additional file 1: Table S1). In addition, among all 3266 participants who had reliable and complete medical records, the association between the monocyte level and the prevalence of DR remained (Additional file 1: Table S2). CRP was marginally associated with the prevalence of DKD. Significant associations of CRP with CVD, ln ACR, eGFR or DR were not found (Additional file 1: Table S3).

## Discussion

In the present study, the results provide evidence about the unique association between monocyte levels and DR. Decreased monocyte levels were associated with increased odds of DR; however, we did not find significant associations between monocyte levels and the prevalence of CVD and DKD or between leukocyte, neutrophil and lymphocyte levels and DR after correction for potential baseline confounders. To the best of our knowledge, previous studies on the association between monocyte levels and DR are almost all basic science studies. Our study is the first large-scale population study about the association between peripheral blood monocyte levels and DR in humans, and the results imply that low peripheral blood monocyte levels may be a biomarker for screening the early stage of DR and could optimize communication between related basic science and clinical studies.

Retinal capillary occlusions, resulting from microvascular thrombi in which leucocytes play a role, have been considered as a characteristic pathologic feature in early DR [26, 27]. In addition, accumulating evidence has suggested that retinal chronic inflammation plays a dominant role in the development of DR [7–9]. Elevated levels of monocytes and neutrophils, caused by the breakdown of the blood-retinal barrier, have been found in the retinal vessels of animals and people with diabetes [10, 14, 27, 28], leading to increased retinal vascular permeability and capillary nonperfusion [13]. Studies have observed activated monocytes, which differentiate into macrophages that secrete cytokines and growth factors and interleukins, adhering to the outer surface of retinal capillaries, resulting in the breakdown of the blood-retinal barrier; the retinal pigment epithelium plays a parallel role, serving as a gateway for monocyte trafficking to the retina following direct or remote injury [13, 14, 29]. The attraction and influx of monocytes into the retina may decrease the monocyte level in the peripheral blood, accounting for our study results, which showed that decreased monocyte levels were associated with increased odds of DR. However, we did not find an association between the level of neutrophils and lymphocytes in the peripheral blood with DR, possibly because there is a complex leukocyte life cycle in humans, where the level of leukocytes in the peripheral blood reaches a balance between the formation of leukocytes, their release from the bone marrow, and their elimination by or recruitment into the tissues [30]. It is worth noting that circulating monocytes are the main cell type entrapped in retinal vessels in patients with DM [31]. The difference between monocyte, neutrophil and lymphocyte cycles across the retina may account for the different associations between monocyte, neutrophil and lymphocyte levels in the peripheral blood and the prevalence of DR.

Interestingly, in the present study, there was no association between monocyte levels and the prevalence of CVD and DKD after adjusting for potential baseline confounders, although increased monocyte levels seemed to be associated with increased odds of CVD and DKD without correction. The reason may be that elevated glucose and obesity, as confounders, could promote the proliferation and activation of monocytes and macrophages with the ability to injure vascular endothelial function in diabetic patients [32–34], which is consistent with our findings. We observed that increased monocyte levels were associated with elevated FPG, HbA1c and BMI, as shown in Table 2. Thus, our findings indicate that HbA1c and BMI may affect the association between monocyte levels and the prevalence of CKD and DKD; however, the association between monocyte levels and the prevalence of DR was independent of HbA1c and BMI, which implies that the association between monocytes and DR is unique and provides evidence for a therapeutic strategy targeting monocyte trafficking for treating DR. The mechanisms explaining the opposite trend in the different diabetic complications are still not clear. We suspect there are two possible reasons. First, different chemotactic responses of monocytes to different organs may account for the different associations between monocyte levels and the prevalence of CKD, DKD and DR [35, 36]. We suspected that the chemotactic responses of monocytes to capillaries of the retina may be stronger than those of heart arteries. Blunted chemotactic responses of monocytes to cardiac arteriogenic stimuli in individuals with DM compared to those without DM, leading to impaired cardiac arteriogenesis [36], increases the susceptibility of diabetic individuals to cardiac events with respect to elevated cardiovascular mortality [37]. However, neovascularization resulting from monocyte aggregation in retinal capillaries is the primary manifestation of PDR, which can lead to acquired blindness [35]. Second, the attraction and influx of monocytes into the retina by adhering to the outer surface of retinal capillaries and breaking down the blood-retinal barrier may decrease the monocyte level in the peripheral blood [13, 14].

In fact, to the best of our knowledge, only one case–control study with 246 patients diagnosed with T2DM, 121 of whom had DR (62 people had NPDR and 59 people had PDR), reported that no significant association was found between monocyte level and DR [38]. The difference in sample size may account for the different results. In addition, to minimize the impact of sample selection, we evaluated the associations between monocyte levels and DR among the participants without proliferative DR (Additional file 1: Table S1) and among all 3266 participants (Additional file 1: Table S2) without excluding the participants treated with laser photocoagulation or intravitreal anti-vascular endothelial growth factor inhibitors, and the results remained consistent.

Although it was an investigation of a large sample of community dwelling participants with strong quality control, there were some limitations in the present study. First, this was a cross-sectional study; thus, causal relationships between monocyte levels and DR cannot be confirmed. The present findings should be cautiously interpreted, and further prospective studies are needed. Second, the prevalence of proliferative DR was low among the participants enrolled in the study, which restricted the extrapolation of the current results to all the stages of DR. Third, although the large sample size of this study has advantages in enhancing the reliability of our findings, a statistically significant finding might not necessarily be clinically significant because of the large sample size in this study; thus, significant findings derived from this study must be further analyzed and interpreted with caution.

## Conclusions

We observed that the decreased level of peripheral blood monocytes was associated with increased odds of DR after adjusting for potential confounders in Chinese adults with DM, which suggests that measuring monocyte levels in a timely manner may be helpful to screen for the early stage of DR. However, the causation between monocytes and DR should be assessed in additional cohort studies.

## Supplementary information

**Additional file 1: Table S1.** Associations between the monocytes level quartiles and the prevalence of DR among the participants without proliferative DR. **Table S2.** Associations between the monocytes level quartiles and the prevalence of DR among all the 3266 participants. **Table S3.** Associations between CRP level and diabetic complications.

## Data Availability

The data supporting the findings of this study are available on reasonable request from the corresponding authors.

## Abbreviations

DM: Diabetes mellitus
DR: Diabetic retinopathy
PDR: Proliferative diabetic retinopathy
NPDR: Nonproliferative diabetic retinopathy
BMI: Body mass index
FPG: Fasting plasma glucose
HbA1c: Glycated hemoglobin
HDL: High-density lipoprotein
LDL: Low-density lipoprotein
CVD: Cardiovascular and cerebrovascular diseases
ACR: Albumin to creatinine ratio
DKD: Diabetic kidney disease
eGFR: Estimated glomerular infiltration rate
MCP-1: Monocyte chemoattractant protein-1
ROC: Receiver operating characteristic
CRP: C-reactive protein level
OR: Odds ratios
CIs: Confidence intervals
