# Supplementary material for: The unique association between the level of peripheral blood monocytes and the prevalence of diabetic retinopathy: a cross-sectional study
Source: J Transl Med. 2020 Jun 22;18:248. doi: 10.1186/s12967-020-02422-9 (PMC7310136; doi:10.1186/s12967-020-02422-9)
Supplement: Supplementary file 1 — Additional file 1: Table S1. Associations between the monocytes level quartiles and the prevalence of DR among the participants without proliferative DR. Table S2. Associations between the monocytes level quartiles and the prevalence of DR among all the 3266 participants. Table S3. Associations between CRP level and diabetic complications. [file 12967_2020_2422_MOESM1_ESM.docx]

|  |  | DR^1^ | DR^2^ |
| --- | --- | --- | --- |
| Monocytes (×10 ^9^ /L) | Q 1(≤ 0.29) | Ref. | Ref. |
|  | Q 2 (>0.29, ≤ 0.36) | 0.94 (0.72, 1.23) | 0.89 (0.68, 1.18) |
|  | Q 3 (>0.36, ≤ 0.44) | 0.92 (0.70, 1.21) | 0.85 (0.64, 1.13) |
|  | Q 4 (> 0.44) | 0.64 (0.48, 0.86) | 0.58 (0.43, 0.80) |
|  | *P* for trend | 0.006 | 0.001 |
|  | per SD increase | 0.87 (0.79, 0.97) | 0.80 (0.72, 0.90) |

Additional Table S1. Associations between the monocytes level quartiles and the prevalence of DR among the participants without proliferative DR

The participants with proliferative DR (n=3) were excluded. 3223 diabetic participants with a mean age of 67 years old (SD 8, min 23, max 99) were involved in the analyses.

Data are expressed as odds ratios (95%CI). Logistic regression analyses were used for the association between the monocytes level and DR.

^1^The model was adjusted for age, sex, duration of diabetes.

^2^The model was adjusted for age, sex, duration of diabetes, education status, current smoking, BMI, HbA1c, dyslipidemia, systolic blood pressure and insulin therapy.

DR, diabetic retinopathy; BMI, body mass index; HbA1c, glycated hemoglobin.

|  |  | DR^1^ | DR^2^ |
| --- | --- | --- | --- |
| Monocytes (×10 ^9^ /L) | Q 1(≤ 0.29) | Ref. | Ref. |
|  | Q 2 (>0.29, ≤ 0.36) | 1.06 (0.81, 1.37) | 1.03 (0.79, 1.34) |
|  | Q 3 (>0.36, ≤ 0.44) | 1.02 (0.78, 1.34) | 0.95 (0.72, 1.26) |
|  | Q 4 (> 0.44) | 0.73 (0.55, 0.98) | 0.68 (0.50, 0.92) |
|  | *P* for trend | 0.040 | 0.012 |
|  | per SD increase | 0.88 (0.79, 0.98) | 0.85 (0.77, 0.95) |

Additional Table S2. Associations between the monocytes level quartiles and the prevalence of DR among all the 3266 participants

All the participants (n = 3266) with a mean age of 67 years old (SD 8, min 23, max 99) who had reliable and complete medical records were involved in the analyses.

2709 (84.1%) participants were diagnosed without DR; 334 (10.2%) participants were diagnosed with DR stage 1; 172 (5.2%) participants were diagnosed with DR stage 2; 35 (1.1%) participants were diagnosed with DR stage 3 and 16 (0.5%) participants were diagnosed with DR stage 4.

Data are expressed as odds ratios (95%CI). Logistic regression analyses were used for the association between the monocytes level and DR.

^1^The model was adjusted for age, sex, duration of diabetes.

^2^The model was adjusted for age, sex, duration of diabetes, education status, current smoking, BMI, HbA1c, dyslipidemia, systolic blood pressure and insulin therapy.

DR, diabetic retinopathy; BMI, body mass index; HbA1c, glycated hemoglobin.

Additional Table S3. Associations between CRP level and diabetic complications

|  | CRP level, ng/ml | | | |  |  |
| --- | --- | --- | --- | --- | --- | --- |
|  | Quartile 1 | Quartile 2 | Quartile 3 | Quartile 4 | *P* for tend | 1SD increment of CRP |
| CVD^1^ | Ref. | 0.90 (0.59, 1.39) | 1.03 (0.67, 1.59) | 0.88 (0.57, 1.36) | 0.709 | 0.92 (0.75, 1.13) |
| lnACR^2^ | Ref. | 0.18 (-0.09, 0.45) | 0.12 (-0.16, 0.40) | 0.13 (-0.15, 0.41) | 0.452 | 0.06 (-0.03, 0.16) |
| DKD^1^ | Ref. | 1.72 (0.98, 3.05) | 2.07 (1.15, 3.72) | 1.78 (0.99, 3.19) | 0.054 | 1.14 (0.96, 1.35) |
| eGFR^3^ | Ref. | -1.66 (-5.26, 1.94) | -2.81 (-6.58, 0.96) | -2.67 (-6.42, 1.08) | 0.132 | -0.23 (-1.48, 1.02) |
| DR^1^ | Ref. | 0.66 (0.35, 1.42) | 0.51 (0.26, 0.99) | 0.70 (0.37, 1.32) | 0.164 | 0.69 (0.36, 1.35) |

We chose one of the communities and measured CRP of all the participants in that community. The number of the participants measuring CRP was 594. The median (interquartile ranges) of CRP in individuals with DR (n=94) and individuals without DR (n=500) was 1.4 mg/L (0.7 mg/L, 2.8 mg/L) and 1.0 mg/L (0.6 mg/L, 2.5 mg/L). No significant difference was found (*P*=0.263).

Data are expressed as regression coefficients or odds ratios (95%CI). Linear regression analysis was used for the association of CRP with lnACR and eGFR respectively. Logistic regression analyses were used for the association of C-reaction protein with CVD, DKD and DR.

^1^The model was adjusted for age, sex, education, duration of diabetes, current smoking, body mass index, HbA1c, dyslipidemia and systolic blood pressure.

^2^The model was adjusted for duration of diabetes, education, current smoking, body mass index, HbA1c, dyslipidemia, systolic blood pressure and eGFR (containing age and sex).

^3^The model was adjusted for education, duration of diabetes, current smoking, body mass index, HbA1c, dyslipidemia and systolic blood pressure.

CVD, cardiovascular and cerebrovascular diseases; DKD, diabetic kidney disease; DR, diabetic retinopathy; ACR, microalbumin to creatinine ratio; eGFR, estimated glomerular infiltration rate; CRP, C-reaction protein.
